# Preservation and Faithful Expression of Transgene via Artificial Seeds in Alfalfa

**DOI:** 10.1371/journal.pone.0056699

**Published:** 2013-05-14

**Authors:** Wenting Liu, Zongsuo Liang, Xinhua Wang, Susan Sibbald, David Hunter, Lining Tian

**Affiliations:** 1 College of Life Sciences, Northwest A & F University, Shaanxi, China; 2 Southern Crop Protection and Food Research Centre, Agriculture and Agri-Food Canada, London, Ontario, Canada; 3 Southern Crop Protection and Food Research Centre, Agriculture and Agri-Food Canada, Vineland Station, Ontario, Canada; University of Nottingham, United Kingdom

## Abstract

Proper preservation of transgenes and transgenic materials is important for wider use of transgenic technology in plants. Here, we report stable preservation and faithful expression of a transgene via artificial seed technology in alfalfa. DNA constructs containing the *uid* reporter gene coding for β-glucuronidase (GUS) driven by a 35S promoter or a tCUP promoter were introduced into alfalfa via *Agrobacterium*-mediated genetic transformation. Somatic embryos were subsequently induced from transgenic alfalfa plants via *in vitro* technology. These embryos were treated with abscisic acid to induce desiccation tolerance and were subjected to a water loss process. After the desiccation procedure, the water content in dried embryos, or called artificial seeds, was about 12–15% which was equivalent to that in true seeds. Upon water rehydration, the dried somatic embryos showed high degrees of viability and exhibited normal germination. Full plants were subsequently developed and recovered in a greenhouse. The progeny plants developed from artificial seeds showed GUS enzyme activity and the GUS expression level was comparable to that of plants developed from somatic embryos without the desiccation process. Polymerase chain reaction analysis indicated that the transgene was well retained in the plants and Southern blot analysis showed that the transgene was stably integrated in plant genome. The research showed that the transgene and the new trait can be well preserved in artificial seeds and the progeny developed. The research provides a new method for transgenic germplasm preservation in different plant species.

## Introduction

Introduction of new traits into plants via genetic transformation has become an important technology for plant improvement. Proper preservation of transgenes and extension of the new traits to the next generation after the plant growth season are important for the wide use of transgenic technology. Herbaceous plants, especially annual plants, grow once a year. At the end of the growth season, plants die and are disposed, and the transgenes can be lost. However, new genes in plants may be stored and preserved in seeds and then passed to the next generation. A transgene in a plant exists in dominant allele status [1]. After self-crossing, a portion of the seeds, specifically, 25% of the seeds, will lose the transgene [2], [3]. Several self-crosses and generations are needed to obtain transgene-homozygous plants and seeds [4]. This can be time and labor consuming. Also, the amount of seeds developed from a plant is often limited. A large number of plants need to be grown to obtain sufficient seeds for research, especially commercial uses. This needs large spaces and land, long periods of time and extensive labor input. In addition, seed development in some plant species is naturally impaired due to various reasons and thus transgenes may not be passed to the next generation and transgenic materials can be lost. Moreover, perennial plants and woody plants need a much longer time to produce seeds. As such, the breeding process to pass transgenes to the next generation in these types of plants can be very slow.

Plants have unique characteristics that allow various cells, after certain induction, to reprogram and develop into somatic embryos [5]–[7]. Somatic embryos have the same morphology and structure as zygotic embryos (seeds) and can germinate and develop into full and fertile plants [8]–[10]. Somatic embryogenesis has been developed in a large number of plant species and the system has been used widely for producing transgenic plants for molecular biology and functional genomics research and in biotechnology for plant trait improvement.

Somatic embryos, after certain treatments such as abscisic acid [ABA], sucrose and heat shock, can acquire tolerance to water loss. They can be dried to contain less than 15% water, similar to the water content in true seeds, and still remain viable under ambient environment. After rehydration, the somatic embryos can germinate and develop into full plants [11]–[15]. Dried somatic embryos can be intact as they are produced or encapsulated and they are collectively called artificial seeds or synthetic seed [16]–[20]. Artificial seeds can be stored for long periods of time and still possess propagation ability. These embryos can be handled or shipped as true seeds. Artificial seeds indeed are a true analog of conventional seeds and can be used for germplasm and genetic material preservation. Artificial seed technology and artificial seed-related technology have been reported in various plant species [12], [14], [15], [20]–[25].

Induction of somatic embryos from transgenic plants and the use of artificial seeds may provide a new system for transgene preservation. Here, we report stable transgene preservation and faithful expression of a transgene in plants developed from dried somatic embryos in alfalfa. The new system can be used to preserve transgenic materials for research use and preserve transgenic germplasm for applications in different plant species.

## Materials and Methods

### The DNA constructs for plant transformation

The *uid* gene coding for β-glucuronidase (GUS) [26] was used as the reporter of transgene expression in the study. The *uid* gene was cloned into pRD400 which was developed from the pBin19 vector [27]. The *uid* gene was either driven by the 2×35S promoter [28] or by the tCUP promoter [29]. The constructs were introduced into *Agrobacterium tumefaciens* GV3101/pMP90 strain by electroporation. The resulting *Agrobacterium* was used for plant transformation.

### Alfalfa plant transformation

GV3101 containing the transformation vectors was cultured in LB (Luria-Bertani) medium containing 100 mg/L rifampicin, 100 mg/L kanamycin and 100 mg/L gentamycin at 28°C until the optical density (OD_600_) reached 0.8–1.0. *Agrobacterium* culture was centrifuged for 8 min at 2500 rpm and the pellets were resuspended in fresh LB medium.

Petioles were dissected to approximately 8 mm in length and were precultured on SH2K medium for two days. SH2K medium consisted of SH salts [30], 50 mM K_2_SO_4_, 25 mM proline, 0.4 mM thioproline [31], 0.56 mM myo-insitol, 0.9 µM kinetin, and 4.5 µM 2, 4-dichlorophenoxyacetic acid (2, 4-D). The explants were then immersed in the bacterial suspension for 4 minutes. After inoculation, the explants were blotted dry on sterile filter paper briefly to remove excess bacterial solution and transferred to SH2K medium supplemented with 20 µM acetosyringone. After two days of co-culture, the explants were transferred and maintained on SH2K medium containing 75 mg/L kanamycin at a two week subculture interval for callus induction and development. Antibiotic resistant calli were selected and transferred to BOi2Y medium [32] supplemented with 75 mg/L kanamycin for embryo development. Developed somatic embryos were transferred to MSO medium containing 75 mg/L kanamycin for germination. MSO medium contained MS medium salts [33] supplemented with 1 mg/L glycine and 2% sucrose. Plantlets were transferred to ½ MSO medium in Magenta boxes for further development.

All culture media were solidified with 0.25% gelrite and adjusted to pH at 5.8 before autoclaving at 121°C for 25 min. Tissue cultures were maintained at 25°C in 54∼72 µmol·m^−2^·s^−1^ white light with 16/8 h light and dark photoperiods.

### Induction of somatic embryos from transgenic plants

Somatic embryos were induced from different and independent transgenic plants using petioles as explants. The procedure was the same as the transformation method but without *Agrobacterium* infection. Cotyledonary-staged somatic embryos of different transgenic alfalfa lines were randomly divided into two groups. One group was used for desiccation treatment and the other was used as controls.

### Desiccation treatment of transgenic somatic embryos

Desiccation tolerance of embryos was induced by application of exogenous ABA [13]. Cotyledonary-staged embryos were maintained on BOi2Y medium supplied with 10 mM ABA for 2 weeks. Embryos treated by ABA were placed on a filter paper wetted slightly with sterile water and the filter paper with the embryos was placed in Petri dishes (60×20 mm) without sealing. The embryos were subjected to desiccation by transferring Petri dishes through a series of six desiccators. The relative humidity (RH) in each desiccator was kept constant by saturated solutions of K_2_SO_4_ (RH 87%), Na_2_CO_3_ (RH 87%), NaCl (RH 75%), NH_4_NO_3_ (RH 63%), Ca (NO_3_)_2_·4H_2_O (RH 51%) and K_2_CO_3_·2H_2_O (RH 43%) in the desiccator [12], [13], [34]. A relative humidity meter was placed in each desiccator and embryos were transferred to the next desiccator when the RH in the desiccators had reached the required level as indicated by the RH meter. The embryos were left in the final desiccator for one additional week to ensure a complete desiccation effect.

### Acclimatization of recovery plants

Embryos with and without desiccation treatments were transferred to ½ MS medium for germination. The resulting plantlets with well-developed root systems were washed with tap water and transplanted into pots containing a 3∶1 mixture of commercial substrate and perlite. The pots were covered with transparent polyethylene bags and sprayed with water to maintain a high relative humidity around the plantlets during the early state of plant adaptation. The relative humidity was gradually reduced in the following two weeks via progressively removing the bags daily. The bags were completely removed and the plants were grown in standard greenhouse conditions two weeks after the plants were transferred into soil.

### Histochemical and quantative GUS expression analyses

Leaf samples were taken from independent transgenic plants developed from embryos with or without desiccation treatment. Histochemical GUS expression was analyzed following the method as described by Jefferson et al [26]. The tissues of transgenic and control plants were immersed in micro-centrifuge tubes containing freshly prepared X-gluc assay solution. The assay solution contained 0.5 mM potassium ferricyanide, 0.5 mM potassium ferrocyanide, 0.3% (*v/v*) Triton X-100, and 1.92 mM 5-bromo-4-chloro-3-indolyl β -D-glucuronide (X-gluc) in 50 mM phosphate buffer (pH 7.0). The tubes were incubated overnight at 37°C. The assay solution was removed on the following day, and plant tissues were decoloured using ethanol with different gradients. The GUS expression level was preliminarily evaluated via visual observation of the intensity of the blue staining in plant leaf tissues with scale from 1 (weak staining) to 3 (intensive staining).

Fluorometric analysis was conducted to measure GUS enzyme activity in leaf tissues as described by Jefferson et al. [26]. Protein content in the extract was determined spectrophotometrically (595 nm) according to Bradford [35] using a commercially available Bradford Reagent dye (Sigma). Measurements of the enzyme activity were repeated 2–4 times after incubation lasting from 15 min to 24 hours depending on the levels of sample fluorescence. GUS activity was expressed as pM 4-MU per mg protein per minute.

### Molecular analysis to confirm plant transformation

Polymerase chain reaction (PCR) analysis was carried out to analyze plant transformation in randomly selected transgenic lines. DNA was isolated from plants developed from desiccated and not-desiccated embryos of each line using the protocol of Lodhi et al. [36]. Primers used to amplify the *uid* gene were: forward primer, 5′- CGTCCTGTAGAAACCCCAAC - 3′ and reverse primer, 5′-ATTGACCCACATTTGCCGT-3′. The expected fragment length was 300 bp. PCR was conducted in a 50 µL reaction mixture containing 100 ng DNA, 200 µM of each dNTP, 1 µM of each primer, 1 U *Taq* DNA polymerase, 1.5 mM MgCl_2_, and 3 µL of 10× *Taq* DNA polymerase buffer. The conditions for PCR reactions were: 1 cycle at 94°C for 5 min, 35 cycles at 94°C for 30 seconds, 58°C for 30 seconds, 72°C for 45 seconds and a final cycle at 72°C for 30 seconds. DNA from non-transgenic plants was used as the negative controls.

Plants were initially analyzed by PCR. Randomly selected lines were then examined by Southern blotting to analyze the integration stability of the transgene. Ten micrograms of genomic DNA was digested with *EcoR*1 that cuts the T-DNA region once and separated on a 0.8% agarose gel, blotted on nylon membrane (Amersham) and fixed by UV cross-linking. The blot was hybridized with DIG- labeled *nptII* probe. The probe was prepared by PCR that amplified a 0.7 kb fragment of the *nptII* gene following supplier's instructions (Bio-Rad, USA).

## Results and Discussion

**Figure 1:**
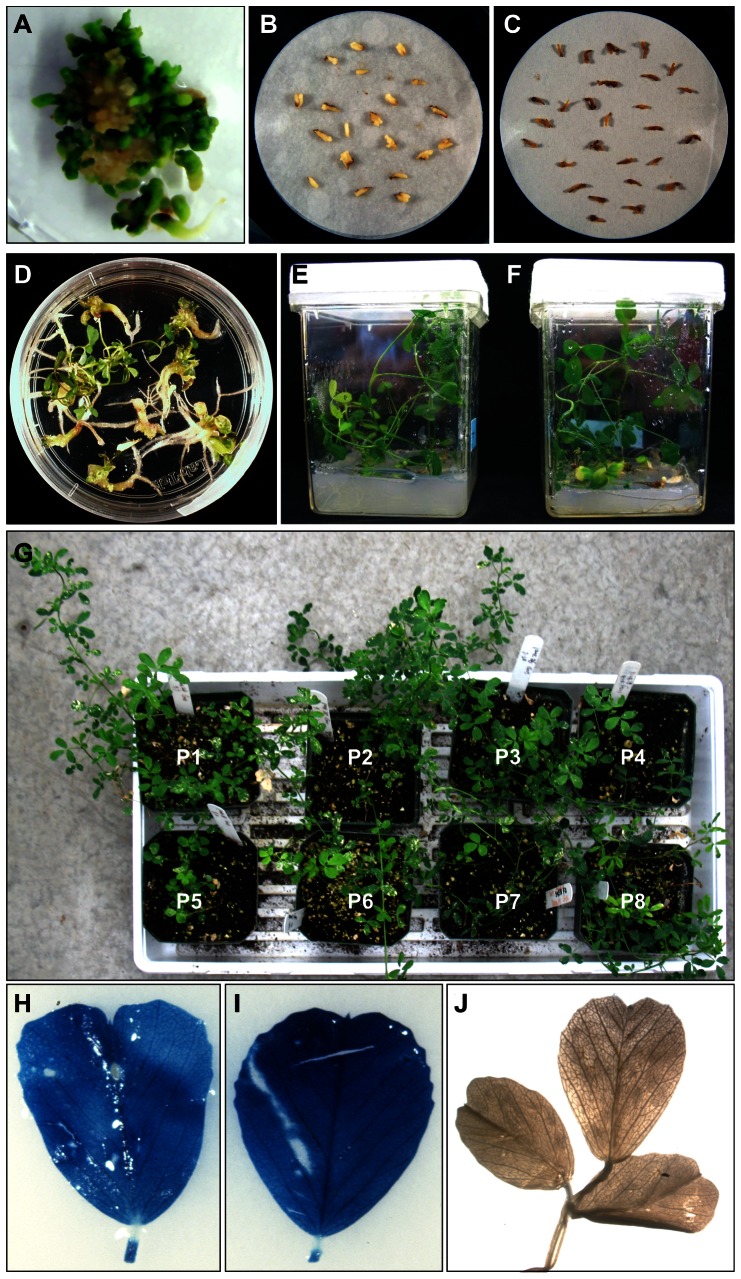
Development and desiccation treatment of somatic embryos induced from transgenic alfalfa plants. A. Development of somatic embryos from a transgenic plant; B. Somatic embryos after ABA treatment. C. Somatic embryos after desiccation treatment. D. Germination of desiccated embryos. E. Development of plants from desiccated somatic embryos. F. Development of plants from somatic embryos without desiccation. G. Recovery of plants from desiccated somatic embryos in a green house. H. GUS expression in a plant developed from an embryo without desiccation; I. GUS expression in a plant developed from desiccated embryo; J. No GUS expression in control plant.

**Figure 2:**
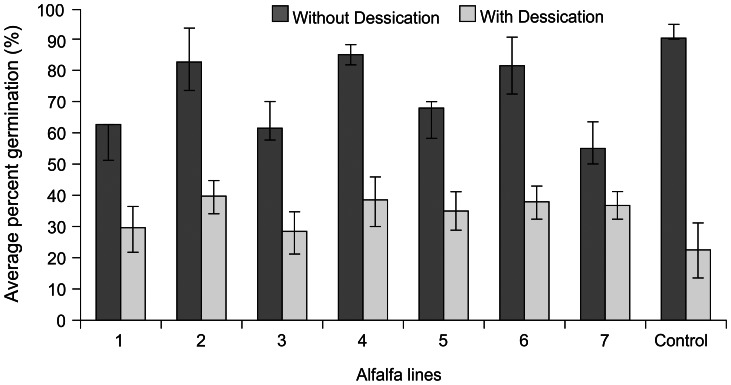
Germination of somatic embryos with and without desiccation treatment of different transgenic alfalfa lines.

Transgenic alfalfa plants were obtained via *Agrobacterium*-mediated transformation using the method well established in our laboratory [37]. Plant transformation was confirmed by PCR using *uid* gene primers, histochemical analysis and Southern blot analysis (described above). Somatic embryos were efficiently induced from different transgenic alfalfa plants (Figure 1A). Freshly induced embryos from transgenic lines contained normal amounts of water. Embryos gradually turned yellow on the BOi2Y medium containing ABA (Figure 1B), which was the sign of acquisition of desiccation tolerance [12], [34]. During the desiccation process, the embryos gradually lost water and the size of the embryos decreased. At the end of desiccation process, somatic embryos had significantly shrank (Figure 1C) and contained only 12–15% water which was equivalent to that in true seeds [12], [13], [34]. The dried somatic embryos were transferred to ½ MS medium for germination and rapidly enlarged to the same size as before desiccation. Within ten days, the embryos turned green and started to produce roots. Shoots subsequently developed and normal plantlets formed (Figure 1D). The average germination rate of somatic embryos subjected to desiccation treatment was 34.8%. This was lower than that of the somatic embryos without desiccation which was 70.8% (Figure 2). Selection of embryos of high quality and optimization of ABA treatment conditions can increase the survival and germination rate of somatic embryos [11], [34], [38]. Overall, a significant percentage of embryos survived the desiccation treatment. On the other hand, the embryos without ABA treatment all lost viability after desiccation and none of the embryos showed germination (not shown). This indicated that transgenic somatic embryos can retain viability as non-transgenic embryos after ABA and desiccation treatments [12], [34].

The rooted plantlets developed from desiccated somatic embryos reached a height of 6∼8 cm within 4 weeks. Plants regenerated from dried somatic embryos were phenotypically the same to those not undergoing the desiccation process (Figure 1E, F). The plants survived and grew well in soil in a greenhouse (Figure 1G).

**Figure 3:**
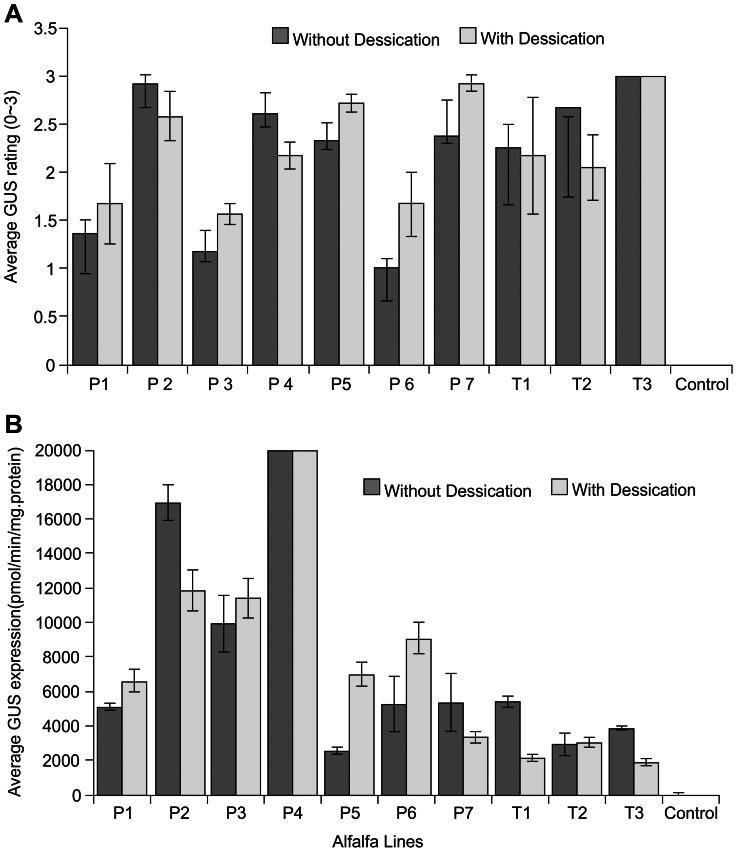
Analysis of GUS expression in plants recovered from embryos with and without desiccation treatment in different transgenic lines. A. Histochemical analysis of GUS expression via visual rating the intensity of the blue staining in plants. B. Fluorometric analysis for GUS expression in plants. P1–P7 were lines transformed with 2×35S-GUS vector; T1–T3 were lines transformed with the tCUP-GUS vector.

GUS expression was analyzed for the plants developed from desiccated embryos and non-desiccated embryos via histochemical assay [26]. Plants developed from desiccated embryos showed intensive GUS staining and the expression level was comparable to that of plants developed from non-desiccated embryos (Figure 1H, I, Figure 3A). GUS expression was further analyzed and quantified via fluorometric assay [26]. High levels of GUS expression were detected in all the plants analyzed (Figure 3B). Different lines showed different levels of GUS expression. Nevertheless, the overall GUS expression patterns among the lines remained the same (Figure 3B). The lines that showed high levels of GUS expression still exhibited high levels of expression after desiccation treatment. Low expression lines still showed low GUS expression after desiccation treatment. The transgene expression level in different lines was determined by other factors, such as the position effect [38]–[40] and was not affected by the desiccation process.

Different transformation constructs were used to evaluate transgene expression. The 35S promoter is a virus derived promoter [28] and has been widely used to drive expression of various genes. The tCUP is plant derived promoter which was identified by promoter-trapping technology [29]. This promoter can drive high levels of gene expression in different plant species and in different organs [37], [41]. Regardless of the promoters used, all of the plants developed from desiccated somatic embryos showed GUS expression and the expression levels were comparable to that developed from embryos without desiccation (Figure 3A, B). Thus, stable expression of the transgene in plants developed from embryo desiccation treatment appeared to be independent of different gene regulatory elements or promoter systems.

**Figure 4:**
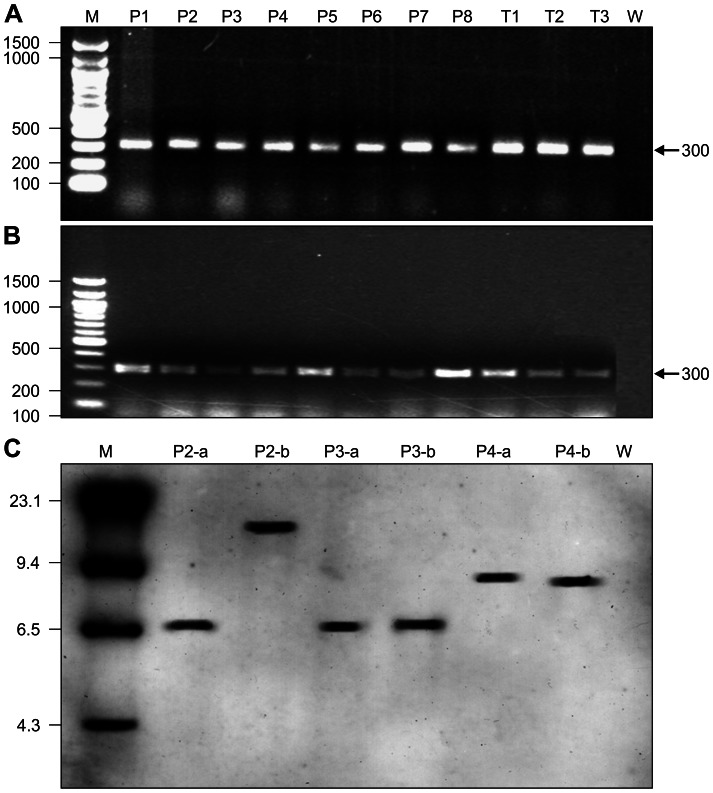
Molecular analysis of transgenic plants. **A.** PCR analysis of plants recovered from embryos without desiccation. B: PCR analysis of plants recovered from embryos subjected to desiccation process. (M):Marker DNA; P1–P8: independent lines transformed with 2×35S-GUS vector; T1–T3; independent lines transformed with the tCUP-GUS vector. (W): Non-transformed control plant. C: Southern blot analysis of different transgenic alfalfa lines developed from embryos without (a) and with (b) desiccation treatment. (M): Marker DNA; P2, P3, P4: Individual lines transformed with 2×35-GUS construct; (W): Non-transformed control plant.

The transgene status in plants was analyzed by PCR and by Southern blot in randomly selected lines with and without embryo desiccation treatment. All of the lines tested were positive for the *uid* gene via PCR analysis (Figure 4A, B), indicating the transgene was present in transgenic plants developed from desiccated embryos. Southern blot was conducted in three randomly selected lines. A single cleavage strategy using *EcoR*1 was applied in hybridization analysis as this method not only can show the copy number of the transgene but more importantly can show the insertion pattern of the transgene in the plant genome. This allows analysis of the structure and status of the transgene in the plant genome before and after desiccation treatment. Southern blot analysis showed that all of the lines developed from embryo desiccation gave hybridization signals (Figure 4C). Thus, the transgene integration in plant genomes remained stable and was not affected by the desiccation process. Different lines had different hybridization patterns as found in other studies [42], [43]. Although the hybridization patterns in P2 and P3 were alike, indeed, these were different and independent transgenic plants. It appeared that all three lines tested had a single copy of the transgene. The positions of the hybridization bands of lines P3 and P4 were identical to that of non-desiccated plants, indicating the transgene physical structure and the relative positions in plant genomes were not affected by the desiccation treatment. The band of line P2, however, appeared to have shifted. The reason for this is not clear. We suspect that it might be caused by technical procedure. However, this line after desiccation treatment still showed a high level of GUS expression. Thus, the gene structure was neither altered nor damaged by the desiccation process.

Genetic integration and genetic presence of transgene in plant genome are in dominant allele status in different plant species. The inheritance of transgene follows the Mendelian genetics that the transgene will segregate when transgenic plants are self-crossed and a quarter of F1 offspring seeds do not carry the transgene. However, transgene homozygous seeds can be obtained via several rounds of crossing and selection and transgene can then be passed to future generations without segregation via conventional seeds. This has been widely studied and documented in different plant species including alfalfa [1]–[4], [44]. Use of artificial seeds which are directly derived from transgenic plants for plant propagation can bypass the traditional breeding process and transgene can be genetically passed to next generation and progenies without segregation. This study has demonstrated this. Indeed, transgene can be passed to progenies indefinitely by artificial seeds. As such, the inheritance of transgene in progeny via conventional breeding was not studied in this report, especially it has been well studied previously [1]–[4], [44].

Artificial seed development is an important as well as a complicated biological process which includes acquisition of desiccation tolerance, significant dehydration, life dominancy and survival of harsh environment and conditions, resuming of various biological programs, and recovery of life processes. It involves in various biochemistry and physiology changes in plants. Although artificial seeds have been reported in various plant species, still, this biological process has not been developed in many other plants because somatic embryos cannot survive dehydration. This indicates the function of many genes have lost during artificial seed development. Thus, studying transgene genetics, especially physical status and the function of transgene in plants developed from artificial seeds is of importance. This study for the first time shows that transgene can be well and stably preserved and transgene expression can be faithfully retained in progenies developed from artificial seeds in a plant species. As no transgene segregation occurs as in true seeds of F1 plants, all of the somatic embryos produced from transgenic plants contain the transgene. Thus the time and labor to select transgenic seeds from F1 plants is not necessary. The dried transgenic somatic embryos containing the new genes and proteins can be easily transported to other locations when needed. In addition, somatic embryo production is much faster compared to seeds and somatic embryos can also be produced in much larger quantity compared to seeds. These features can be useful in various aspects. This research provides a novel and useful technology to produce and maintain transgenic materials for research use and for other usage for the species in which the artificial seed technology has been developed. Along with the research advancement and technology improvement, the artificial seeds will have a wider application in plant research and plant biotechnology.
